# High-resolution imaging as a tool for identifying quantitative trait loci that regulate photomorphogenesis in *Arabidopsis thaliana*

**DOI:** 10.1093/aobpla/plab063

**Published:** 2021-09-24

**Authors:** Stephen D Deslauriers

**Affiliations:** Division of Science and Math, University of Minnesota, Morris, Morris, MN 56267, USA

**Keywords:** High-resolution phenotyping, photomorphogenesis, QTL analysis

## Abstract

A primary component of seedling establishment is the photomorphogenic response as seedlings emerge from the soil. This process is characterized by a reduced growth rate in the hypocotyl, increased root growth, opening of the apical hook and expansion of the cotyledons as photosynthetic organs. While fundamental to plant success, the photomorphogenic response can be highly variable. Additionally, studies of *Arabidopsis thaliana* are made difficult by subtle differences in growth rate between individuals. High-resolution imaging and computational processing have emerged as useful tools for quantification of such phenotypes. This study sought to: (i) develop an imaging methodology which could capture changes in growth rate as seedlings transition from darkness to blue light in real time, and (ii) apply this methodology to single-quantitative trait locus (QTL) analysis using the Cvi × L*er* recombinant inbred line (RIL) mapping population. Significant differences in the photomorphogenic response were observed between the parent lines and analysis of 158 RILs revealed a wide range of growth rate phenotypes. Quantitative trait locus analysis detected significant loci associated with dark growth rate on chromosome 5 and significant loci associated with light growth rate on chromosome 2. Candidate genes associated with these loci, such as the previously characterized *ER* locus, highlight the application of this approach for QTL analysis. Genetic analysis of Landsberg lines without the *erecta* mutation also supports a role for ER in modulating the photomorphogenic response, consistent with previous QTL analyses of this population. Strengths and limitations of this methodology are presented, as well as means of improvement.

## Teaser

Machine vision and computational analysis represents a growing tool set for the study of developmental traits in plant biology. It is particularly valuable for studying dynamic processes in real time. This work presents a methodology for the study of photomorphogenesis using high-resolution imaging and measurement of growth rate with ImageJ software. An 8-h time course, consisting of 3 h in darkness and 5 h of exposure to blue light, is sufficient to resolve significant differences in growth rate in each condition. This methodology can be used for identification of genetic elements that regulate the light response.

## Introduction

Successful establishment of the seedling is a critical determinant of overall plant health. This is dependent on the response of the germinating seed and growing seedling to many internal and external factors. Studies using the model organism *Arabidopsis thaliana* have provided significant insight into the role conditions such as temperature and light play in establishment (Franklin *et al.* 2014).

The response to light is characterized by a number of phenotypic changes in seedling growth and development (Gommers and Monte 2018). During skotomorphogenesis, etiolated seedling hypocotyls grow at an accelerated rate, root growth is limited, the cotyledons form into a protective apical hook structure and limited amounts of chlorophyll are synthesized (Wei *et al.* 1994; Leivar *et al.* 2009; Sassi *et al.* 2012). Upon perception of light, seedlings undergo photomorphogenesis, which is characterized by a significantly reduced rate of growth in the hypocotyl, increased root growth rate, opening of the apical hook, expansion of cotyledon tissue and production of chlorophyll (Josse *et al.* 2011; Raz and Koornneef 2001; Toledo-Ortiz *et al.* 2010; Galvão and Fankhauser 2015; Chen *et al.* 2016). While this sequence of developmental changes has been well-characterized, the process itself can be highly variable on an individual basis. Additionally, environmental factors such as light intensity, light quality and elevated temperatures have all been shown to modulate aspects of photomorphogenesis. (Koussevitzky *et al.* 2007; Bou-Torrent *et al.* 2014; Jung *et al.* 2016). Identification of genetic elements in *Arabidopsis* that can be associated with variability in the photomorphogenic response would be of significant value to the study of seedling establishment overall.

Quantitative trait locus (QTL) analysis of *A. thaliana* mapping populations has been remarkably successful at identifying regions of the genome which are important for multifactorial traits in development (Alonso-Blanco *et al.* 2009). Crossing two parental lines with differing phenotypic values, followed by repeated self-crossing of the progeny, leads to a large mapping population with unique—and known—combinations of the parental DNA. The Cvi × L*er* population of recombinant inbred lines (RILs) generated by Alonso-Blanco *et al.* (1998a) has been utilized for analysis of flowering time, seed dormancy and seed size to name a few (Alonso-Blanco *et al.* 1998a, 2003; Moore *et al.* 2013a). This population has also been utilized to identify loci important for the response to white, blue, red and far-red light (Borevitz *et al.* 2002; Botto *et al.* 2003). Both Borevitz *et al.* and Botto *et al.* used manual measurement of hypocotyl length and apical hook angle in response to various dark and light conditions.

Recent advances in computational imaging have allowed for measurement of subtle phenotypes in high resolution (Spalding 2009). These systems have been particularly effective at tracking changes over time, such as root tip angle during the course of a gravitropic response (Miller *et al.* 2010). Such an approach would be ideal for the study of photomorphogenesis, specifically the changes in growth rate which accompany detection of blue light. A time course consisting of initial growth in the dark, followed by growth in the light, can be carried out without disturbing the seedlings for measurement. Importantly, image resolution would be sufficiently high to track changes in hypocotyl length <0.1 mm. Small changes of this nature have made it challenging to study dynamic properties of the photomorphogenic response in *Arabidopsis*. Machine visioning can also be implemented in a high-throughput manner so as to efficiently screen through larger mapping populations, demonstrated by QTL analysis of the root gravitropic response in the Cvi × L*er* RIL population (Moore *et al.* 2013b).

This project sought to apply high-resolution imaging techniques to QTL analysis of the photomorphogenic response in the Cvi × L*er* RIL population, with a particular emphasis on growth rate. The rationale is similar to work conducted by Borevitz *et al.* (2002) in that both studies sought to analyse hypocotyl growth in darkness and blue light, but important differences in the methodology for this study are as follows: (i) growth rate was determined over relatively short time periods rather than overall hypocotyl length after 4 days, (ii) hypocotyl length was determined through computational measurement rather than by hand, (iii) growth in darkness and blue light was assessed within the same experimental trial in order to monitor dynamic changes in response to light and (iv) single-QTL modeling was used rather than multiple-QTL modeling. Strengths and limitations of this methodology are presented, as well as the identification of loci important to growth in the dark and in blue light.

## Methods

### Plant material and growth conditions

The Cvi × L*er* population of *A. thaliana*, constructed and genotyped by Alonso-Blanco *et al.* (1998a, b), was used in this study. Seeds were provided by Dr Edgar Spalding from University of Wisconsin–Madison. La(ER) seeds were provided by Arabidopsis Biological Resource Center (ABRC) at the Ohio State University. Seeds were surface-sterilized and cold-treated at 4 °C for 96 h for stratification. Seeds were then sown on agar plates containing 1 mM KCl, 1 mM CaCl_2_, 5 mM 4-morpholineethanesulfonic acid, pH 5.7 with BIS-TRIS propane, and gelled with 1 % agar. Germination was induced by exposure to white light for 1 h. Plates were then wrapped in foil for etiolated growth and incubated vertically at 23 °C for 96 h.

### Imaging

All images were captured using a fixed Marlin F146B camera (Allied Vision Technologies), fitted with a macro zoom lens (model NT59-157; Edmund Optics). Media plates were positioned and secured vertically in a custom-designed acrylic holder and backlit with 880 nm infrared light for imaging. This is similar to the imaging apparatus described in Moore *et al.* (2013b) but this work utilized a single camera rather than multiple cameras in series. Specifications for the hardware and set-up instructions for the imaging apparatus are freely available through phytomorph.wisc.edu. Imaging took place in a dark room kept at ~23 °C. Green lighting was used for manipulation of the seedlings and positioning of plates for imaging.

### Hypocotyl growth rate measurement

ImageJ software (v 1.52k; https://imagej.nih.gov/ij/) (Rasband 1997) was used to determine growth rate of seedlings. A metric scale bar was included on media plates during imaging to determine a fixed calibration value of 35.509 pixels per mm. Midlines were traced manually using the segmented lines function of ImageJ to measure hypocotyl length for seedlings at each time point.

### QTL analysis

Significant loci associated with each phenotype were determined using the r/qtl2 package (Broman *et al.* 2018) for RStudio statistical software (v. 4.0.5; RStudio Team 2020; http://www.rstudio.com/). Association was based on genotype information for the RIL population constructed by Alonso-Blanco *et al.* (1998a) using 293 amplified fragment length polymorphism (AFLP) -based markers (Alonso-Blanco *et al.* 1998b). Marker data and genetic mapping information were obtained from The Arabidopsis Information Resource (TAIR). Pseudomarkers were inserted at 1-cM intervals and a genotyping error rate of 0.001 was assumed, as described in Broman and Sen (2009). This package uses the hidden Markov model to account for missing genotype information. The genome was then scanned using a one-dimensional, single-QTL model with Haley–Knott regression (Haley and Knott 1992) using 25 000 permutations of genotype for each phenotype. Significance thresholds of *α* = 0.05 and 0.01 were used.

## Implementation

**Figure 1.**
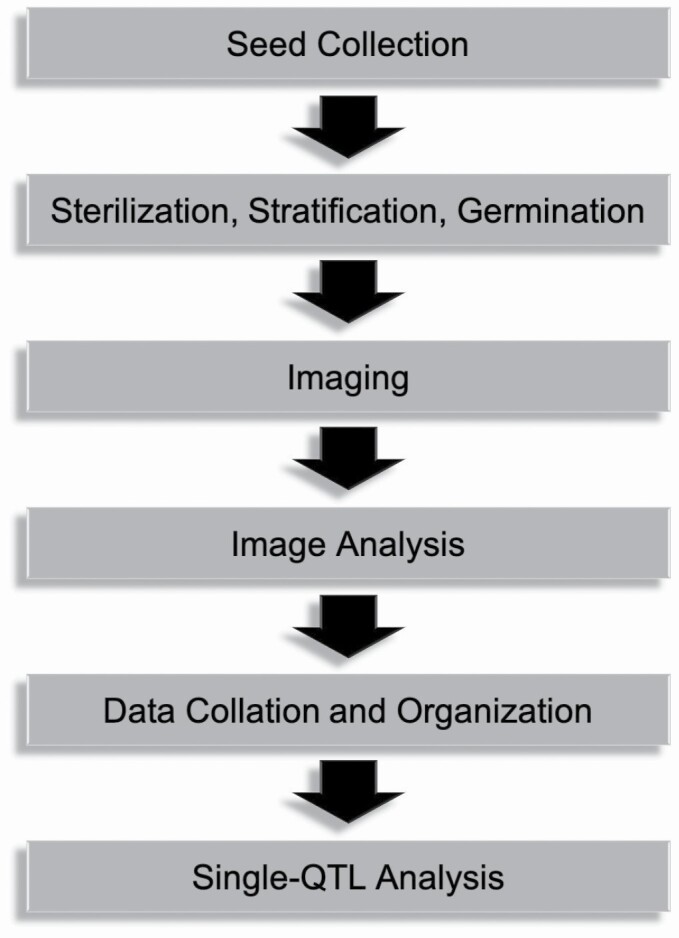
Workflow. A summary of the project implementation is shown.

A work flow for capturing and analysing growth rate phenotypes was carried out as follows and is summarized in Fig. 1:

1. Seed collection: Fresh, viable seeds are required for each parent (Cvi and L*er*) as well as each of the 162 Cvi × L*er* RILs. A supply of 100 seeds of each should be sufficient so long as they germinate efficiently. Growth rate data should be collected from as many RILs as possible to increases the confidence of the QTL analysis performed later.
2. Sterilization, stratification, and germination of seeds:
  - Seeds should be surface-sterilized according to whichever protocol is preferred; this study used the following procedure:
    ◦ Transfer ~20–30 seeds to a microcentrifuge tube.
    ◦ Add 200 µL of 70 % Ethanol.
    ◦** Wash**: Add 200 µL diH_2_O. Vortex, then centrifuge at 10 000 rpm for 30 s. Remove the supernatant, being careful to leave seeds on the bottom of the tube.
    ◦ Repeat wash step three more times.
    ◦ Add 100 µL of bleach and incubate at RT for 5 min. Periodically vortex the seeds in order to resuspend them.
    ◦ Repeat wash step four times.
  - Seeds can be immediately stratified after sterilization by wrapping the tube in foil and storing at 4 °C for 96 h.
  - Seeds can be germinated by plating on Minimal Media (1 mM KCl, 1 mM CaCl_2_, 5 mM 4-morpholineethanesulfonic acid, pH 5.7 with BIS-TRIS propane; 1 % agar), and exposing to white light for 1 h. Each media plate should contain ~20 seeds of a single genotype. Seal plates with Millipore tape to allow for air exposure and prevent contamination. To assess etiolated growth rate, wrap media plates in foil before incubating at 23 °C for 96 h. Plates should be incubated vertically to promote seedling growth on the agar surface and easier imaging.
3. Imaging: Based on the imaging platform available for this study, the following procedure was carried out:
  - Transfer foil-wrapped seedlings to a dark room before removing the foil. Green light can be used without affecting the seedling responses.
  - Carefully position seedlings in the middle of the plate in a single row, such that 10 or more seedlings are within a 5-cm space, with no seedlings touching. Also include a ruler of known length as a scale bar for at least one image.
  - Open the camera imaging software and capture an image of each media plate for time point 0 (*T*_0_). The apical hooks must be in frame and remain in frame throughout the 8-h time course.
  - Reseal media plates with Millipore tape and wrap in foil, then return seedlings to 23 °C growth chamber. Incubate vertically in the same orientation as the original gravitational vector for 3 h.
  - Transfer foil-wrapped seedlings back to the dark room and remove the foil. Position media plates for seedling imaging and capture image files for time point 1 (*T*_1_).
  - Place the seedlings under 20 µM m^−2^ s^−1^ blue light (470 nm) for 5 h.
  - Return seedlings to the dark room for imaging and capture images for time point 2 (*T*_2_).
  - Transfer all image files to an external drive or cloud system for analysis.
4. Image Analysis: Midline tracing with ImageJ can be used to determine hypocotyl length and growth rate according to the following procedure:
  - Setting the scale:
    ◦ Using the straight or segmented line feature, draw a line along the ruler or scale bar. Any known length is sufficient.
    ◦ Use Command M (Mac OS) to measure the length of this line in pixels.
    ◦ From the top menu, select ‘Analyze’, then ‘Set Scale’. Input the measurement from above as distance in pixels, input the known distance in mm and check ‘global’ to apply this scale calibration to all following images for analysis.
  - Tracing the midline:
    ◦ Select the segmented lines feature from straight line icon dropdown.
    ◦ Add points to trace the hypocotyl of a seedling, following the midline as closely as possible.
    ◦ After placing the last point, right click to stop drawing the line and adjust any points so that the trace follows the hypocotyl midline as closely as possible.
    ◦ Use Command M to measure the midline, giving the hypocotyl length in mm.
  - Determining growth rate:
    ◦ For dark growth rate (**DGR**), use the following formula:

$$\frac{\text{Hypocotyl ​​}\;\text{​ Length ​}\;\text{​ }T^{1}\;-\;\text{​ Hypocotyl ​}\;\text{​ Length ​}\;\text{​ }T^{0}}{3\text{ ​}\;\text{​ h}}$$ (1)

◦ For light growth rare (**LGR**), use the following formula:

$$\frac{\text{Hypocotyl ​}\;\text{​ Length ​}\;\text{​ }T^{2}\text{ ​}\;\text{​ }-\text{ ​}\;\text{​ Hypocotyl ​}\;\text{​ Length ​}\;\text{​ }T^{1}}{5\text{ ​}\;\text{​ h}}$$ (2)

◦ For Percent Reduction in growth rate, use the following formula:

$$1-\frac{LGR}{DGR}$$ (3)

5. Data Collation and Organization: Average growth rate and percent reduction data for Cvi, L*er* and each RIL must be organized into a single spreadsheet that can be called in RStudio for QTL analysis. The first column should contain genotype information, with subsequent columns containing phenotype data.
6. Single-QTL analysis: Quantitative trait locus analysis of growth rate and percent reduction in growth rate data was carried out using the r/qtl2 package developed by Broman *et al.* (2018) in RStudio. For an in-depth guide, the user is referred to Broman and Sen (2009); a list of the available functions in r/qtl2 and their output is also available at kbroman.org/qtl2.

## Results and Discussion

**Figure 2.**
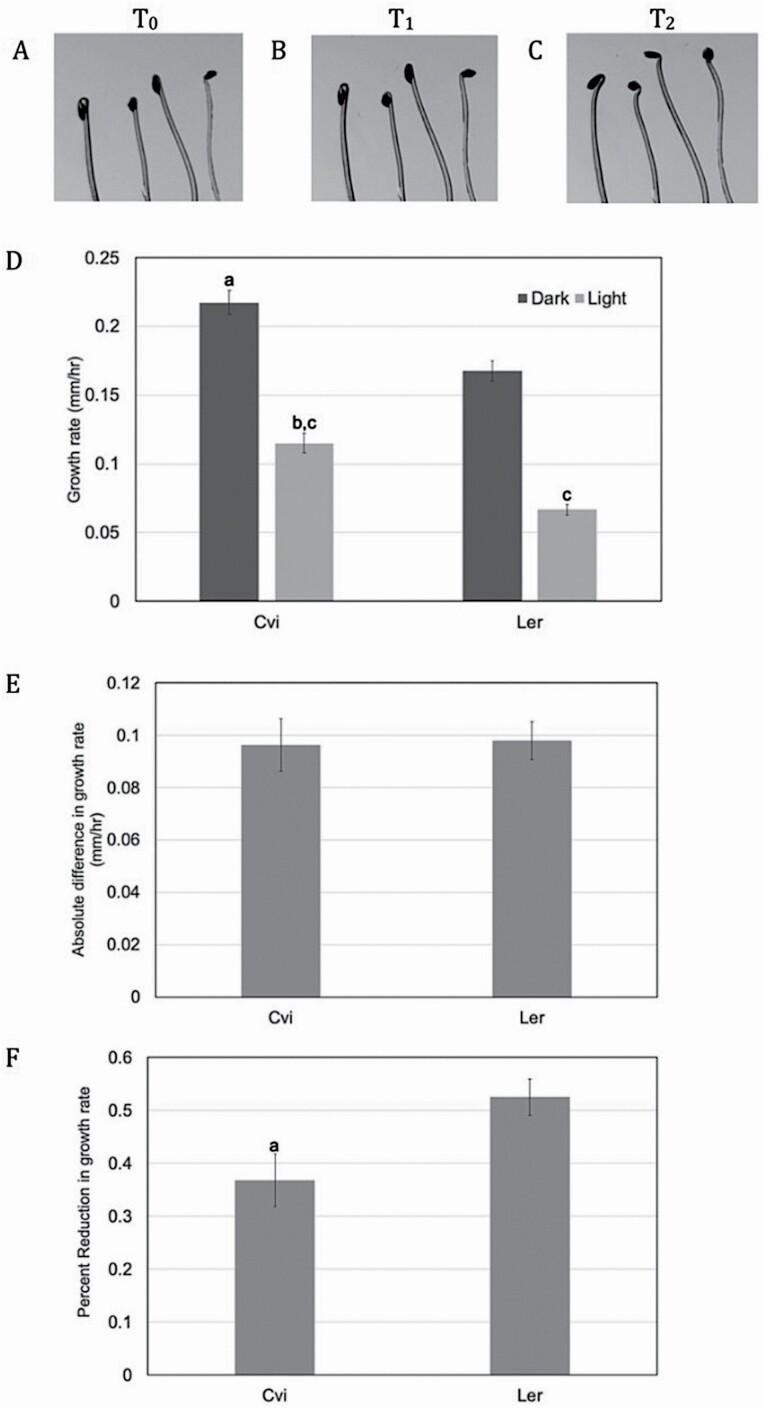
Imaging approach and growth rate analysis in Cvi and L*er*. Representative images of 3-day-old Cvi seedlings taken at (A) an initial time point (0 h) following repositioning, (B) after 3 h of growth in the dark (3 h dark) and (C) after 5 h of growth in 20 µM m^−2^ s^−1^ blue light. Hypocotyl length was determined using midline tracing in ImageJ software. (D) Growth rates (mm h^−1^) for each parent line were determined using hypocotyl length data from images taken at each time point. *a* represents *P* < 0.001 between L*er* and Cvi dark growth rates, *b* represents *P* < 0.001 between L*er* and Cvi light growth rates and *c* represents *P* < 0.001 between dark and light growth rates within each parent line. (E) Absolute difference in growth rate was determined by subtracting the growth rate in the light from the growth rate in the dark. (F) Percent reduction following exposure was determined by subtracting the ratio of light growth rate:dark growth rate from 1.0. *a* represents *P* < 0.01. Mean ± SE was determined from 64 L*er* seedlings and 54 Cvi seedlings.

The first goal of this project was to establish a methodology that would be suitable for measuring the photomorphogenic response in *A. thaliana*. Reduction in hypocotyl growth rate following exposure to blue light was chosen as an indicator of the response. Using a single-camera set-up in a dark room, seedlings grown vertically on minimal media were imaged using an infrared light source. To compare light and dark growth rates, at least three time points are required for imaging: following repositioning (*T*_0_), after a period of growth in the dark (*T*_1_) and after a period of growth in blue light (*T*_2_) (Fig. 2). Time intervals needed to be sufficiently long to allow for measurable amounts of growth in the hypocotyl in each condition. Furthermore, blue light needed to be used at a low enough fluency to allow for the hypocotyl to detectably grow, but grow at a significantly slower rate than in darkness. After experimentation, it was determined that 3 h of growth in the dark (Fig. 2B) and 5 h of growth under 20 µM m^−2^ s^−1^ blue light was effective (Fig. 2C) for capturing the photomorphogenic response. Due to the availability of a single camera for imaging in the dark room and the desire for high-throughput data collection, seedlings could not be left in front of the camera for the duration of the imaging time course. Rather, multiple plates with unique seed lines were set up and individually positioned for imaging at each time point. This approach allowed for rapid screening of multiple independent seed lines, though a disadvantage was that positioning an individual seed line for imaging was rarely identical across all three time points. Therefore, computational measurement of hypocotyl length was difficult to automate and growth rate was determined from manual analysis through ImageJ software (v 1.52k; https://imagej.nih.gov/ij/). ImageJ (Rasband 1997) has been widely used for quantification of plant structures and phenotypes. Vasseur *et al.* (2018) applied this software for prediction of plant dry mass based on measurable features in the leaf. ImageJ has also been applied to quantification of hypocotyl curvature in response to tropic stimuli (Yamamoto and Haga 2019).

Initial experiments conducted on the parent lines illustrate the difference in response between the Cvi and L*er* ecotypes grown in darkness and in light. As previously demonstrated by Botto *et al.* (2003), Cvi seedlings were significantly longer than L*er* in both dark and light conditions. A significantly higher growth rate for Cvi was also observed in both conditions. As expected, both ecotypes displayed significantly reduced growth rate in response to blue light (Fig. 2D). To quantify the photomorphogenic response, the absolute difference in growth rate as well as percent reduction following exposure to light were determined. Comparison of the absolute difference in growth rate in each condition did not reveal a significant difference between Cvi and L*er* (Fig. 2E). However, L*er* seedlings displayed a significantly higher percent reduction in growth following exposure to blue light (Fig. 2F), which is explained by their lower growth rate in the dark compared to Cvi and suggests that this aspect of the photomorphogenic response is exaggerated in L*er* backgrounds. Percent reduction in growth rate was therefore used as an indicator of the photomorphogenic response moving forward.

**Figure 3.**
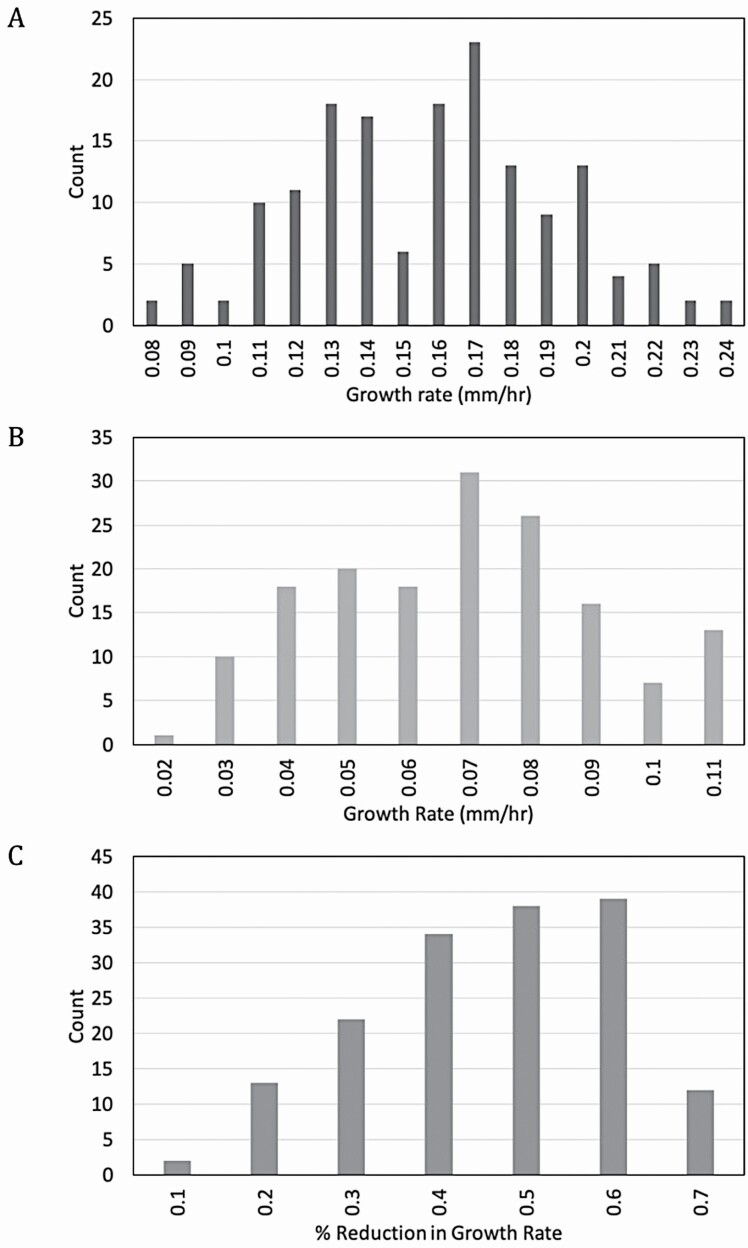
Distribution of growth rate phenotypes in Cvi × L*er* RILs. Dark growth rate (A), light growth rate (B) and percent reduction in growth rate following exposure to blue light (C) were determined for 158 RILs as described in Fig. 1. Frequency distributions of each are shown. *N* ranged from 3 to 23 seedlings for each RIL, with an average of 11.7 seedlings per RIL.

Growth rate analysis was then extended to the population of Cvi × L*er* RILs generated by Alonso-Blanco *et al.* (1998a) and used by Borevitz *et al.* (2002) to identify genetic loci associated with the response to a range of light qualities. Of the 162 RILs, data were collected from 158 members of the population, resulting in distributions with a wide range of growth rate phenotypes (Fig. 3). In darkness, the average growth rate was 0.161 mm h^−1^ ± 0.003 (Fig. 3A). In blue light, the average growth rate was 0.073 mm h^−1^ ± 0.001 (Fig. 3B). The average percent reduction in growth after exposure to blue light was 51.2 % ± 0.01 (Fig. 3C).

Standard analysis of variance (ANOVA) indicates that phenotypic variation between the RILs can be explained by variation in the genotype (Table 1). MS_M_ (mean square model) reflects variance due to genotypic differences between RILs while MS_E_ (mean square error) reflects variance due to environmental factors. At a *P-*value of 0.001, the critical *F*-value for all three phenotypes is 1.41. The calculated *F*-value for each phenotype exceeds the critical value, rejecting the null hypothesis. Therefore, variance in each phenotype can be attributed to genotypic variation between the RILs. Heritability for each phenotype is also shown (Table 1) and was calculated with the r/qtl2 package developed by Broman *et al.* (2018). While the growth rate phenotypes each display a modest level of heritability, this data set predicts very little heritability for the percent reduction in growth rate phenotype.

**Table 1.** Heritability of growth rate phenotypes. MS_M_, mean square model; MS_E_, mean square error; df_M_, degrees of freedom for model; df_E_, degrees of freedom for error. ***P < 0.001.

Trait	MS_M_	MS_E_	df_M_	df_E_	*F*-value	Heritability
Dark growth rate	1.11 × 10^−2^	3.36 × 10^−3^	158	1730	3.32***	0.428
Light growth rate	7.63 × 10^−3^	1.06 × 10^−3^	158	1730	7.16***	0.52
Percent reduction in growth rate	2.38 × 10^−1^	9.81 × 10^−2^	158	1730	2.43***	0.236

**Figure 4.**
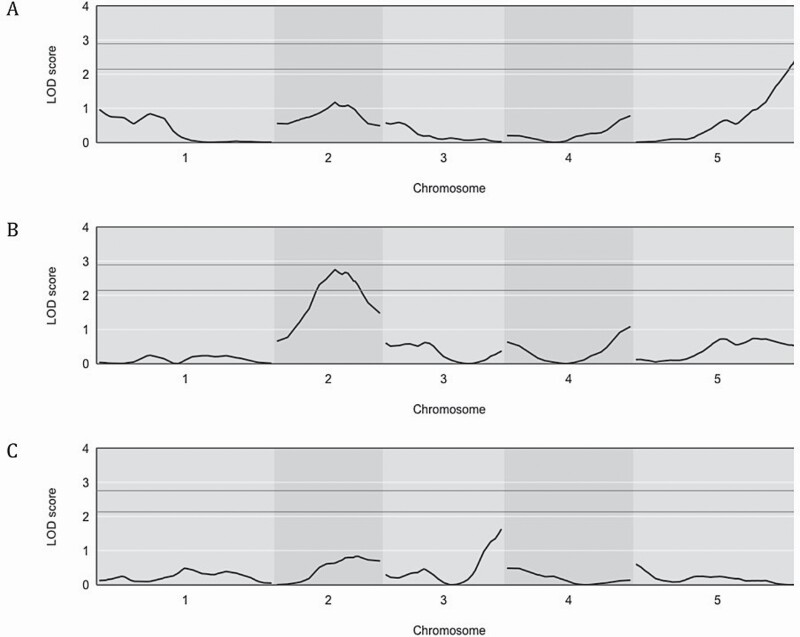
LOD profile of genetic association with photomorphogenic phenotypes. Quantitative trait locus analysis was carried out for dark growth rate (A), light growth rate (B) and percent reduction in growth rate following exposure to blue light (C) using the Haley–Knott regression feature of the r/qtl2 library (Broman *et al.* 2018) in R statistical software. Logarithm (base 10) of odds (LOD score) on the *y*-axis represents the likelihood that a locus is associated with the phenotype of interest. Positions along each chromosome (in cM) are on the *x*-axis. The horizontal lines represent the LOD significance threshold of 2.4 (*P* < 0.05) and 2.7 (*P* < 0.01).

Quantitative trait locus analysis of growth rate and percent reduction data was performed using interval mapping and single-QTL modeling in the r/qtl2 package. Analysis was based on the use of 293 markers spanning the *Arabidopsis* genome, producing Logarithm of Odds (LOD) scores for each phenotype (Fig. 4). These LOD scores indicate the likelihood of association of the phenotype with each chromosomal marker. They were determined using 25 000 permutations of the genotype for each phenotype. For dark growth rate, significant loci were discovered on chromosome 5. The highest LOD value of 2.59 appeared at 113 cM (Fig. 4A). This locus was not associated with hypocotyl length in the dark by Borevitz *et al.* (2002), which could be explained by the differences in approach. Significant loci for light growth rate were also identified on chromosome 2 ranging from 34 to 54 cM. The highest LOD value of 2.75 appeared at 40 cM (Fig. 4B). Quantitative trait locus analysis of percent reduction in growth rate in response to blue light did not reveal a locus with an LOD value over the 2.4 significance threshold (Fig. 4C). The locus with the highest LOD value (1.61) appeared on chromosome 3 (80 cM). Of particular interest is a locus on chromosome 2 (59 cM) with an LOD value of 0.785, which overlaps somewhat with the broad significant QTL identified for growth rate in the light. Further analysis may strengthen confidence for this locus being associated with both phenotypes.

**Figure 5.**
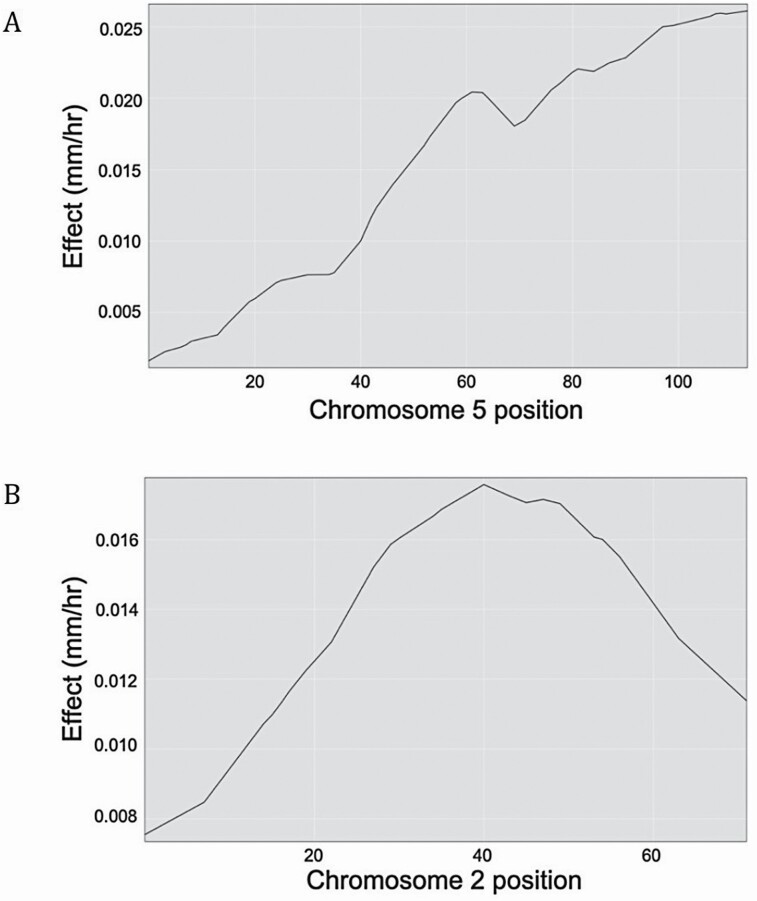
Estimated effect of parent alleles on growth rate. The estimated effect of the Cvi versus L*er* parent allele on growth rate (mm h^−1^) is shown for the dark growth rate QTL identified on chromosome 5 (A) and the light growth rate QTL identified on chromosome 2 (B). AA represents the effect of L*er* alleles at each position on the chromosome while BB represents the effect of Cvi alleles at each position. Positive values for QTL effect indicate that the allele has a positive effect on growth rate. Negative values indicate a negative effect on growth rate.

Quantitative trait locus peaks for dark growth rate and light growth rate were fairly broad and could be explained by a number of different factors. One possibility could be due to a high level of natural phenotypic variation within the individual seed lines. It could also be that the multiple marker regions within these loci contribute small individual effects, which cumulatively account for the difference between the two parent lines. While single-QTL models are limited in their analysis of potential additive effects or epistatic interactions, they have reliably identified significant loci associated with a specific phenotype (Broman and Sen 2009). Quantitative trait locus effects for the dark growth rate and light growth rate phenotypes are shown in Fig. 5. For both phenotypes, the presence of a Cvi allele resulted in a positive effect on growth rate.

Analysis of genes within the identified loci supports the approach used in this study. A list of candidates for each locus is shown in Table 2. An obvious stand-out from this list is *ERECTA (ER)*. Effects of this loss-of-function mutation in the commonly used Landsberg ecotype are wide ranging, including a number of developmental and physiological pathways (Van Zanten *et al.* 2009). In particular, adult plants are somewhat shorter in the L*er* background (Rédei 1962) and produce fewer seeds (Torii *et al.* 1996). *ER* has also been shown to modulate auxin-dependent cell elongation (Woodward *et al.* 2005). Quantitative trait locus analysis of this same RIL population revealed that the *ERECTA* locus (47–53 cM) can be associated with hypocotyl growth in darkness, blue light and response to the BR inhibitor Brassinazole (BRZ) (Borevitz *et al.* 2002), as well as shade avoidance (Kasulin *et al.* 2013).

**Table 2.** Candidate genes for dark growth rate and light growth rate QTL. Genes associated with the significant loci and their described roles are indicated. Markers and genes are listed in sequential order.

Dark growth rate—5@113 cM		
Associated markers	LOD value	Position
FD.188C	2.59	113 cM
HH.122C/120L	2.59	113 cM
EG.205L	2.59	113 cM
Candidate genes	Role	
AT5G67030 (ABA1)	ABA biosynthesis; hypocotyl growth during skotomorphogenesis (Barrero *et al.* 2008)	
AT5G67100 (ICU2)	Regulation of SA and ABA responses (Micol-Ponce *et al.* 2015)	
AT5G67160 (EPS1)	SA biosynthesis (Torrens-Spence *et al.* 2019); suppression of hook formation (Huang *et al.* 2020)	
AT5G67250 (SKIP2)	F-box protein; auxin responses (Schwager *et al.* 2007)	
AT5G67440 (NPY3)	PIN localization (Furutani *et al.* 2011)	
AT5G67530 (PUB49)	Plant U-box protein, E3 ligase, unknown role	
Light growth rate—2@40–49 cM		
Associated marker	LOD value	Position
CH.1500C	2.48	34 cM
BF.221L	2.53	35 cM
FD.85C	2.75	40 cM
GB.105L-Col	2.66	43 cM
FD.150C	2.61	45 cM
GD.460L-Col	2.67	47 cM
CC.332C	2.67	47 cM
Erecta	2.65	49 cM
GPA1	2.65	49 cM
CH.145L-Col/150C	2.65	49 cM
AD.191L	2.42	53 cM
BH.195L-Col	2.42	53 cM
GD.298C	2.41	54 cM
Candidate genes	Role	
AT2G23460 (XLG1)	Regulation of cytokinin responses (Wang *et al.* 2017)	
AT2G3550 (MES6); AT2G3560 (MES7); AT2G3570 (MES19); AT2G3580 (MES4); AT2G3590 (MES8); AT2G3610 (MES3); AT2G3620 (MES1)	Methyl esterase family, auxin homeostasis (Yang *et al.* 2008)	
AT2G24180 (CYP71B6)	Auxin homeostasis (Böttcher *et al.* 2014)	
AT2G24540 (AFR)	F-box protein, phytochrome-mediated light responses (Harmon and Kay 2003); photomorphogenesis (Aguilar-Hernández *et al.* 2017)	
AT2G24610 (CNGC14)	Auxin-induced changes in root growth (Shih *et al.* 2015)	
AT2G24790 (COL3)	COP1-interacting protein, photomorphogenesis (Datta *et al.* 2006)	
AT2G5160 (CYP82F1)	Cytochrome p450 family member, unknown role	
AT2G5170 (PKL/EPP1)	Repression of photomorphogenesis (Jing *et al.* 2013); BR-gibberellin integration, skotomorphogenesis (Zhang *et al.* 2014)	
AT2G5180 (RR1)	Cytokinin signalling, light responses (Argyros *et al.* 2008)	
AT2G25490 (EBF1)	F-box protein, degradation of PIF3, photomorphogenesis (Dong *et al.* 2017)	
AT2G25700 (SK3)	E3 ligase, regulation of phytochrome-A-dependent light responses (Marrocco *et al.* 2006)	
Candidate genes	Genes	
AT2G25930 (ELF3)	Vegetative photomorphogenesis and floral induction (Zagotta *et al.* 1996); shade avoidance and hypocotyl growth (Coluccio *et al.* 2011)	
AT2G26000 (BRIZ2)	E3 ligase, germination and seedling growth (Hsia and Callis 2010); skotomorphogenesis (Clouse and Sasse 1998)	
AT2G26070 (RTE1)	Regulation of ethylene responses (Zhou *et al.* 2007)	
AT2G26330 (Erecta)	Auxin-dependent cell elongation (Woodward *et al.* 2005); hypocotyl growth in darkness and blue light (Borevitz *et al.* 2002)	
AT2G26670 (HY1)	Phytochrome biosynthesis (Muramoto *et al.* 1999); photomorphogenesis (Davis *et al.* 2001).	
AT2G26700 (PID2)	PIN localization, phototropism (Haga *et al.* 2014)	

**Figure 6.**
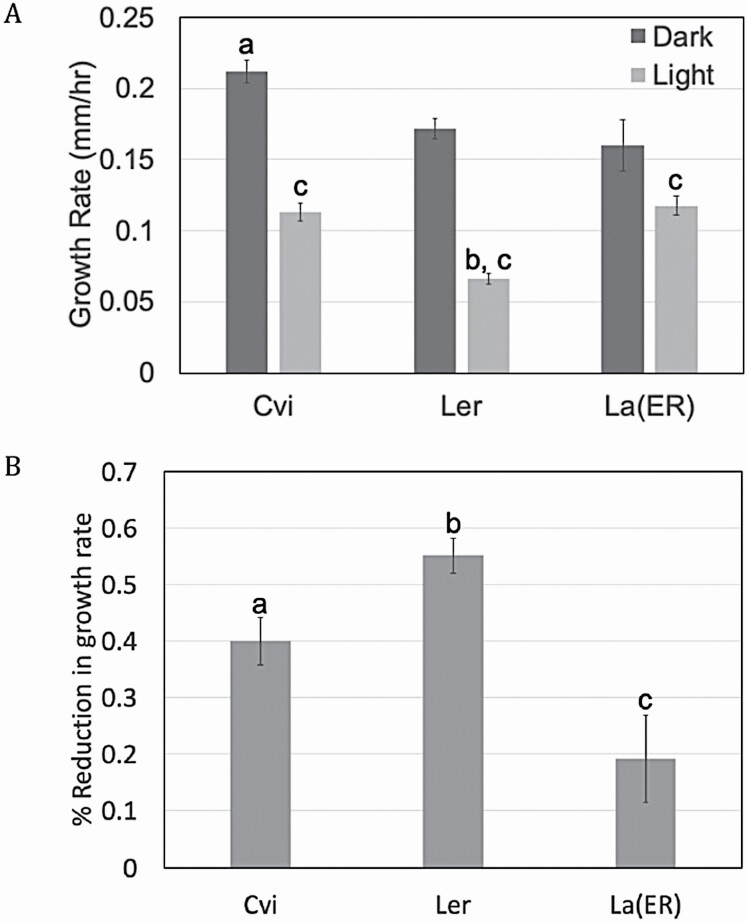
Genetic analysis of ER in light responses. (A) Growth rates (mm h^−1^) for each parent line were determined using hypocotyl length data from images taken at each time point. *a* represents *P* < 0.01 between Cvi and both L*er* and La(ER) dark growth rates, *b* represents *P* < 0.001 between L*er* and La(ER) light growth rates and *c* represents *P* < 0.05 between dark and light growth rates within each parent line. (B) Percent reduction following exposure was determined by subtracting the ratio of light growth rate:dark growth rate from 1.0. *a* represents *P* < 0.01 between Cvi and L*er*, *b* represents *P* < 0.001 between L*er* and La(ER) and *c* represents *P* < 0.05 between La(ER) and Cvi. Mean ± SE was determined from 64 L*er* seedlings, 54 Cvi seedlings and 11 La(ER) seedlings.

Genetic analysis was carried out to determine if *ER* plays a significant role in the photomorphogenic response. Using the same conditions described above (Figs 2 and 3), Landsberg seedlings homozygous for wild-type *ER* (La(ER)) were analysed and growth rate was determined. As shown in Fig. 6, dark growth rate in La(ER) seedlings was significantly lower than Cvi, but surprisingly did not significantly differ from L*er* (Fig. 6A). While Borevitz *et al.* (2002) indicate that the *er* allele resulted in shorter hypocotyls in the dark in both Landsberg and Columbia backgrounds, the difference is marginal and could easily be explained by the small differences in growth rate between L*er* and La(ER) shown in Fig. 6. The data presented here suggest that *ER* plays a limited role in seedling growth rate in the dark and cannot explain the observed significant difference between Cvi and L*er*. Consistent with this, Shpak *et al.* (2004) demonstrated that the *er* mutation is associated with a reduced rate of cell proliferation, rather than cell elongation. Elongation has been shown to be the primary driver of hypocotyl growth in darkness (Wei *et al.* 1994). The *ER* locus also did not appear to be significantly associated with the dark growth rate phenotype in this analysis (Fig. 4A). It should be noted that the single-QTL method of analysis used in this study considers each locus independently, rather than jointly considering multiple QTL together. This is in contrast to the method used by Borevitz *et al.* which performed multiple-QTL modeling and showed a clear association between the *ER* locus and hypocotyl length in darkness. The difference in modelling may account for why the *ERECTA* locus did not appear to be associated with dark growth rate in this analysis. It is also important to note that this study tracked growth rate over a 3-h time period in 4-day-old seedlings, whereas Borevitz *et al.* measured overall hypocotyl length following 4 days of growth. Developmental timing is another possible explanation for the observed differences in role for *ERECTA* in dark grown seedlings.

In contrast, growth rate of La(ER) seedlings in blue light supports a role for *ERECTA* in the blue light response. La(ER) seedlings grew at a significantly faster rate in blue light compared to L*er*, and were not significantly different from Cvi seedlings (Fig. 6A). Of particular interest to the photomorphogenic response, the percent reduction in growth rate following exposure to blue light was significantly less in La(ER) compared to both Cvi and L*er* seedlings (Fig. 6B). This can be explained by the observation that while La(ER) seedlings grow significantly more slowly than Cvi in the dark, their growth rate following exposure to blue light is not significantly different. This is in contrast to L*er*, which is significantly slower than Cvi in both conditions (Fig. 6A). While the blue light response is undoubtedly regulated by a wide number of genes, the observed differences between L*er* and La(ER) support a role for ER in modulating this response, consistent with what has been shown by Borevitz *et al.* (2002).

As further support of the strength of this approach for QTL analysis, several genes within the loci associated with skotomorphogenesis are involved in hormone activity or homeostasis (Table 2). A role for auxin in this response is not surprising given its well-characterized activity in promoting hypocotyl elongation in etiolated seedlings (Rayle and Cleland 1992). The association of SKIP2 and NYP3, which are responsible for modulating auxin response and PIN localization, respectively, supports this single-QTL approach. *PUB49* also appears at this locus. The Plant U-box (PUB) family of proteins have recently been shown to have activity as E3 ligases (Wang *et al.* 2017), which serve a number of important roles in regulating PIF activity and hormone signalling during development. PUB49 may be worth further study in relation to skotomorphogenesis. Other candidate genes for association with the dark growth rate phenotype include factors involved in abscisic acid (ABA) and salicylic acid (SA) responses. These hormones have not been characterized as key regulators of seedling establishment, but Barerro *et al.* (2008) have described a role for the ABA biosynthesis enzyme ABA1 in hypocotyl growth during skotomorphogenesis. Similarly, EPS1, which is involved in SA biosynthesis (Torrens-Spence *et al.* 2019), has recently been associated with suppression of apical hook formation (Huang *et al.* 2020). Another candidate gene at this locus, ICU2, has been shown to regulate the responses of ABA and SA (Micol-Ponce *et al.* 2015). Accordingly, the roles of ABA and SA in seedling establishment may be more significant than previously known.

Candidate genes that may be associated with the light growth rate phenotype are also shown in Table 2. Standouts include *HY1*, which has been demonstrated to be required for photomorphogenesis (Davis *et al.* 2001), and *ERECTA*. Other genes within the loci identified on chromosome 2 have demonstrated roles in the photomorphogenic response, such as *AFR*, *COL3*, *PKL*, *RR1*, *EBF1*, *SK3*, *ELF3*, *BRIZ2* and *PID2* (Zagotta *et al.* 1996; Datta *et al.* 2006; Marrocco *et al.* 2006; Argyros *et al.* 2008; Hsia and Callis 2010; Coluccio *et al.* 2011; Haga *et al.* 2014; Jing *et al.* 2013; Aguilar-Hernández *et al.* 2017; Dong *et al.* 2017). *PKL* and *BRIZ2* are of particular interest due to described roles in skotomorphogenesis as well (Clouse and Sasse 1998; Zhang *et al.* 2014). Other candidate genes for analysis include those which regulate hormone activity or homeostasis but have not yet been associated with photomorphogenesis. These include *XLG1*, which regulates cytokinin responses (Wang *et al.* 2017); a family of methyl esterases (*MES*) which may control auxin levels (Yang *et al.* 2008); *CYP71B6*, which is involved in auxin homeostasis (Böttcher *et al.* 2014); *CNGC14*, which is connected to auxin-induced changes in root growth (Shih *et al.* 2015); *CYP82F1*, which encodes a cytochrome p450 family member of unknown function; and *RTE1*, which regulates ethylene responses (Zhou *et al.* 2007).

Of particular note, genes such as *CRY1*, *CRY2*, *PHYA*, *PHYB*, *COP1*, *HY5*, and other factors known to be involved in seedling establishment were not significantly associated with either growth rate phenotype in this study. This may be due to limitations in the imaging and measurement system. Natural phenotypic variability within the individual RILs also may have reduced confidence in identifying those loci as significant. Variance in the light growth rate and percent reduction in growth rate phenotypes were particularly high and may indicate variation in sensitivity to light. Such phenotypic plasticity has been demonstrated in a large number of early-flowering *Arabidopsis* mutants (Pouteau *et al.* 2004). Individual seedling exposure to light in this experimental set-up may also have been impacted by neighbouring seedlings.

In terms of other QTL analyses which have made use of this same population, seed size is of particular interest (Moore *et al.* 2013a). Since the energy necessary for seedling growth is initially derived from storage molecules in the seed, it is reasonable to expect that growth rate and seed size may share QTLs. Indeed, some loci, such as *ERECTA*, were significantly associated with seed area by Moore *et al.* and growth rate in the light as described above.

## Conclusions

The imaging methodology described herein represents a valuable opportunity to examine specific features of the photomorphogenic response in high resolution. The work is closely related to analyses conducted by Borevitz *et al.* (2002), but has focused on small changes in hypocotyl growth rate that are characteristic of seedling development in response to light rather than overall hypocotyl length. This study demonstrates that high-resolution imaging is capable of detecting small differences in growth rate between genetically unique *Arabidopsis* seedlings under various conditions. Furthermore, single-QTL analysis identifies genetic loci which can be significantly associated with subtle phenotypes like growth rate and candidate genes within these loci have previously been associated with traits related to seedling growth. The challenge lies in the method of measurement.

Previous studies have taken advantage of software tools such as HYPOTrace or customized software for extraction of seed or seedling features (Wang *et al.* 2009; Miller *et al.* 2010; Moore *et al.* 2013a, b; Spartz *et al.* 2017). These methods allow for automated measurements of growth rate that are likely to be more precise than manual, user-determined measurements. The need to reposition plates throughout the time course in this project resulted in seedlings changing position in each image. Computational measurement of hypocotyl length was therefore difficult to automate and manual measurements were made as accurately as possible. Limitations in manual measurement may account for the reduced confidence in locus identification. The 293-marker set used in this study is the most recent AFLP marker library available for the Cvi × L*er* RIL population. It has been successfully used to identify significant QTL in this RIL population (Alonso-Blanco *et al.* 1998a, b, 2003; Borevitz *et al.* 2002; Botto *et al.* 2003; Moore *et al.* 2013a, b) and it is therefore unlikely that the analysis was hindered by the chosen marker set.

Refinement of the imaging system could greatly improve QTL analysis of features related to photomorphogenesis. Inclusion of multiple high-resolution cameras would eliminate the need for removing or repositioning plates for imaging. The entire time course could then be carried out simultaneously on multiple RILs, which would not only allow for automation of measurement but would also support the inclusion of many more time points, and, where necessary, additional data points for lines with low germination rates. Extraction of growth-related features at such a high resolution could reveal the timescale in which photomorphogenic changes take place. A similar approach has been used to identify genetic loci associated with root gravitropism and how the roles of those loci change over the 8-h time course of root tip reorientation (Moore *et al.* 2013b). It is likely that a similar analysis would reveal significant loci associated with features of the photomorphogenic response.

Analysis of apical hook opening, while challenging to measure in this study, also represents a valuable point of comparison to hypocotyl growth rate as an indicator of the photomorphogenic response. The methodology described above would require modification, as seedlings would need to be positioned in a way to prevent obstructions to hook opening. Further studies will seek to examine the sensitivity and variability of both growth rate and apical hook opening to blue light in a genetic context.

## Supporting Information

The following additional information is available in the online version of this article—

1. All images used for determination of growth rate and percent reduction in growth rate.

2. Data sheet summarizing dark growth rate, light growth rate, and percent reduction in growth rate for every seedling measured for this study.

These materials can be accessed at the following link: https://doi.org/10.5281/zenodo.5535053

plab063_suppl_Supplementary_Data

## Data Availability

In the interest of replication of growth rate analysis, development of image processing and statistical mapping methods, all data for this work are included as **Supporting Information**. Data and seedling images used for analysis, as well as all figure image files are also freely available at the following link: https://doi.org/10.5061/dryad.47d7wm3f0.

## Notes

**Phenome, Genome & Environment. Chief Editor**: Colleen Doherty
